# Awareness of Menopause and Health Risks Among Reproductive-Age Women in Latvia

**DOI:** 10.3390/jcm15166295

**Published:** 2026-08-14

**Authors:** Jānis Jurkāns, Jeļizaveta Kolosovska, Elizabete Ārgale

**Affiliations:** Department of Obstetrics and Gynaecology, Riga Stradins University, LV-1007 Riga, Latvia

**Keywords:** menopause, menopause awareness, cardiovascular risk, reproductive health, women’s health, cross-sectional survey, health literacy, Latvia

## Abstract

**Background/Objectives**: The menopausal transition is a period of increasing cardiometabolic risk and a window for prevention, yet preparation is often delayed until symptoms appear. Latvia has a high cardiovascular mortality burden, but population-specific menopause-awareness data are limited. This study aimed to describe item-level awareness among women aged 18–49 years in Latvia, compare age groups, and explore associated factors. **Methods**: An exploratory, anonymous, cross-sectional online convenience survey included 431 women aged 18–49 years in Latvia recruited through paid social media posts. An investigator-designed 47-item Latvian questionnaire was used. Primary outcomes were item-level responses to menopause-awareness Likert statements, summarized using medians, interquartile ranges, and dichotomized evidence-aligned proportions. A study-specific 10-item composite awareness score was constructed post hoc as a secondary hypothesis-generating measure. Analyses used chi-square tests, nonparametric tests, and exploratory multivariable linear regression. **Results**: Recognition of hot flushes (93.0%), that symptoms may appear before age 50 (91.6%), and vaginal dryness (79.8%) was high, whereas awareness that menopause may increase cardiovascular risk was limited (50.3%). Nine out of ten items showed no significant age-group difference; one reverse-keyed memory item showed a small nominal association. Composite awareness scores did not differ by age group, education level, or gynaecologist visit frequency. Internal consistency was modest (Cronbach’s α = 0.688), and the regression model explained limited variance (*R*^2^ = 0.083; adjusted *R*^2^ = 0.052). Previous information-seeking (*B* = 1.85, 95% CI 0.79–2.95, *p* < 0.001) and underweight BMI (*B* = −5.91, 95% CI −8.96 to −2.85, *p* < 0.001; *n* = 12) were independently associated with the composite awareness score. Overall, 72.6% considered educating younger women important. **Conclusions**: In this exploratory convenience sample, strong symptom recognition coexisted with limited cardiovascular-risk awareness, supporting earlier menopause education in Latvia that integrates cardiometabolic prevention.

## 1. Introduction

Menopause is a universal life transition, yet preparation for it is frequently delayed until symptoms disrupt daily life [1]. This reactive approach may leave many individuals uneducated and underprepared, although symptoms can occur years before the final menstrual period [1,2]. The awareness gap matters clinically because midlife is a critical and often overlooked prevention window [3]. Cardiovascular risk accelerates during the menopausal transition, and major scientific statements emphasize early, proactive risk recognition and counselling [3]. Yet a significant gap persists between guideline recommendations and patient awareness [1].

Evidence suggests that many individuals reach midlife with limited menopause knowledge, often reporting minimal education before age 40 [4]. This lack of preparation creates uncertainty about the normality of symptoms and the use of evidence-based interventions [1]. While recent data highlight menopause as an emerging public health issue, the factors shaping awareness in reproductive-age individuals remain understudied [5,6].

In Latvia, population-specific data to inform educational strategies are limited. Latvia is a high-income country in Northern Europe with a population of approximately 1.84 million at the beginning of 2025 [7]. Despite its economic status, the country faces significant public health challenges, including one of the lowest life expectancies in the European Union, estimated at 76.4 years in 2024 [8]. Furthermore, Latvia reports a disproportionately high burden of cardiovascular disease, with mortality rates from circulatory system diseases reaching nearly twice the European Union average [9].

To address this gap, we conducted an exploratory online cross-sectional survey of Latvian women aged 18–49 years. The primary aim was to describe item-level menopause awareness and compare evidence-aligned responses between age groups. Secondary hypothesis-generating analyses examined a study-specific composite awareness score and factors associated with awareness, menopause information-seeking, and previous physician discussion.

## 2. Materials and Methods

### 2.1. Study Design

An exploratory cross-sectional, anonymous, online survey was conducted among women aged 18–49 years residing in Latvia between September 2024 and February 2025. Although reproductive age is commonly defined as 15–49 years, this study included only adult women aged 18–49 years because minors were not eligible to participate. The study used a non-probability convenience sampling approach. The questionnaire was administered using Google Forms and distributed via paid Facebook posts targeting women aged 18–49 across different regions of Latvia. Access was distributed through a Facebook link; however, further sharing of the link outside the platform could not be technically excluded. Eligibility was based on self-reported age, sex/gender and residence in Latvia.

No randomization of items or adaptive questioning was applied. No technical measures such as IP tracking, cookies, or login restrictions were implemented to prevent multiple submissions. The survey invitation post received 3046 views according to Facebook metrics. This metric does not represent unique eligible individuals or actual survey access, and data on the number of unique individuals who clicked through to the Google Form were unavailable. Therefore, participant flow from Facebook post views to form access could not be quantified, and a conventional response rate could not be calculated. Before starting the survey, mandatory informed electronic consent was obtained. No incentive was offered for participation. The study was reviewed and approved by the Research Ethics Committee of Riga Stradins University (approval No. 2-PĒK-4/386/2024) on 3 May 2024. It was reported in accordance with the Checklist for Reporting of Survey Studies (CROSS) and Checklist for Reporting Results of Internet E-Surveys (CHERRIES) for web-based survey reporting [10,11].

### 2.2. Participants

In total, 557 responses were obtained. After excluding 126 respondents younger than 18 or older than 49 years, 431 participants were included in the analysis. All survey questions were mandatory; therefore, no partially completed questionnaires were recorded. Participants were divided into two age groups: 18–39 years and 40–49 years. This allowed comparison of younger women with those more likely to be approaching perimenopause or menopause, as natural menopause typically occurs between the ages of 45 and 55 years and perimenopause may begin in the early 40s.

### 2.3. Survey Instrument and Measurement

This investigator-designed survey was developed specifically for the present study based on the study objectives and English-language survey questionnaires described in previous studies [4,12,13,14,15]. The study authors, all native Latvian speakers, translated and adapted relevant concepts from these sources into Latvian. The resulting mandatory 47-item survey covered sociodemographic and anthropometric characteristics, lifestyle and health-related habits, gynaecologic care and menstrual status, menopause awareness, symptom recognition, perceived health risks, information-seeking, communication with healthcare professionals, and beliefs concerning menopause management and education. Most items were reformulated as study-specific questions, with wording and response options modified for the present survey; additional items were developed by the authors. No cited questionnaire was reproduced or administered as an intact instrument. Before distribution, certified gynaecologists who were native Latvian speakers reviewed the questionnaire for clinical accuracy, content relevance, and clarity of Latvian wording. No formal forward–back translation, formal content-validity assessment, structured cross-cultural adaptation, cognitive pretesting, pilot testing, or pre-administration psychometric validation was performed. The questionnaire took approximately 10 min to complete.

The primary outcome measures were item-level responses to 5-point Likert-scale statements assessing awareness of menopause-related concepts, symptoms, and selected health risks, with responses ranging from 1 (completely disagree) to 5 (completely agree). These items addressed the definition of menopause, recognition of menopausal symptoms, and selected associated health risks, and were used in the primary awareness analyses. Additionally, items on menopause management beliefs were analysed separately as secondary descriptive outcomes and were not included in the awareness outcome measure.

For item-level descriptive reporting, Likert-scale responses were dichotomized as evidence-aligned or non-evidence-aligned to estimate the proportion of participants actively endorsing a response consistent with current evidence and to enable uniform presentation across positively and reverse-keyed items. For positively keyed items, scores of 4–5 were classified as evidence-aligned; for reverse-keyed items, disagreement with the original statement represented the evidence-aligned response. These items were reverse-coded (1→5, 2→4, 3→3, 4→2, and 5→1) before calculation of the composite awareness score and item-level presentation. For presentation, their statement labels were correspondingly presented in the evidence-aligned direction so that higher displayed values consistently represented greater awareness. Neutral responses (score of 3) were grouped with non-evidence-aligned responses because they did not demonstrate active endorsement of the evidence-consistent position, though they were not interpreted as definitively incorrect. These thresholds were based on item content and the meaning of the response categories rather than on the observed response distribution. Dichotomization was restricted to item-level percentages and group comparisons. For item-level medians and interquartile ranges, the two reverse-keyed items were reverse-coded so that higher values consistently represented greater menopause awareness across all items. These consistently oriented five-point scores were also used to calculate the exploratory composite awareness score.

As a secondary hypothesis-generating analysis, a study-specific composite menopause-awareness score was constructed post hoc using 10 items selected from the menopause-related item pool within the 47-item questionnaire; the individual items are reported in Section 3.2. Candidate items were identified based on their relevance to three content domains: understanding of menopause, recognition of menopause-related symptoms, and awareness of potential health risks.

The final item set was selected primarily to improve internal consistency, as assessed using Cronbach’s α, while maintaining conceptual representation across all three domains. Items unrelated to these domains were excluded conceptually, while additional candidate awareness items were not retained when their inclusion reduced the internal consistency of the score. Because item selection and internal-consistency assessment were conducted using the same sample, the score was not prespecified and is subject to selection-related bias and potentially optimistic internal-consistency estimates.

Two reverse-keyed items concerning memory improvement and improved libido were included in the questionnaire with the intention of reducing acquiescent responding and indicating whether participants differentiated item content rather than agreeing indiscriminately. These items were not validated attention checks and were not used to exclude participants. Their responses were reverse coded before the ten item scores were summed to produce a total ranging from 10 to 50. Consequently, higher displayed values consistently indicated greater evidence-aligned awareness. Exploratory factor analysis was subsequently performed to describe the dimensional structure of the finalized item set but was not used as an item-selection criterion.

The composite awareness score was developed solely to summarize overall response patterns and generate hypotheses rather than serve as a validated psychometric instrument. Primary interpretation was therefore based on individual item-level responses, while the composite awareness score was interpreted strictly as a secondary exploratory measure.

### 2.4. Statistical Analysis

Categorical participant characteristics were summarized using frequencies and percentages. Differences between age groups in higher education, BMI category, physical activity level, and menstrual/menopausal status were examined using Pearson’s chi-square tests, with Cramér’s *V* reported as the corresponding effect-size measure. For item-level awareness analyses, the original five-point Likert responses were summarized using medians and interquartile ranges, while evidence-aligned responses were summarized using frequencies and percentages. Differences in dichotomized evidence-aligned responses between age groups were examined using Pearson’s chi-square tests, with Cramér’s *V* reported as the corresponding effect-size measure. Items assessing self-perceived information about menopause-related symptoms and the perceived relevance of educating younger women about the menopausal transition were treated as secondary descriptive outcomes and summarized using frequencies and percentages. Differences between age groups in these outcomes were assessed using Pearson’s chi-square tests with Cramér’s *V*.

After construction of the finalized 10-item composite awareness score, its internal consistency within the present sample was described using Cronbach’s α. Exploratory factor analysis using principal axis factoring was subsequently performed to examine the dimensional structure of the finalized item set. The Kaiser–Meyer–Olkin measure and Bartlett’s test of sphericity were used to evaluate the suitability of the data for factor analysis. Factor retention was assessed using eigenvalues greater than 1.0 together with visual inspection of the scree plot. These analyses were considered exploratory and were not treated as validation of the composite score.

The Shapiro–Wilk test indicated that the composite awareness score was not normally distributed. Mann–Whitney *U* tests were therefore used for comparisons between two groups, and Kruskal–Wallis tests were used for comparisons involving three or more groups. When a Kruskal–Wallis test was significant, Bonferroni-adjusted pairwise comparisons were performed as appropriate. Effect sizes were reported as *r* = |*Z*|/√*N* for Mann–Whitney *U* tests and epsilon-squared (ε^2^) for Kruskal–Wallis tests. Participants who selected “unable to assess” were excluded from the comparison of composite awareness scores by self-perceived information level. Additional exploratory Mann–Whitney *U* tests compared composite-score values according to reported sources of menopause information.

In all multivariable linear and logistic regression models, including the menstrual-status sensitivity models, categorical predictors with k categories were represented using k − 1 dummy variables. The composite awareness score was modelled as a continuous outcome in an exploratory multivariable linear regression. The model included age group, education, previous discussion about menopause with a physician, previous menopause information-seeking, BMI category, physical activity level, and frequency of gynaecologist visits. Normal BMI, no physical activity, and annual or more frequent gynaecologist visits served as the respective reference categories. Regression coefficients were reported with 95% confidence intervals. Model diagnostics included examination of the residual distribution and assessment of multicollinearity using tolerance and variance inflation factors. Category-specific sample sizes were considered when interpreting estimates from sparsely represented predictor categories.

Separate exploratory binary logistic regression models were used to examine factors associated with previous menopause information-seeking and previous discussion about menopause with a physician. Both models included age group, education, BMI category, physical activity level, and frequency of gynaecologist visits. Physician discussion was included as a predictor in the information-seeking model, while information-seeking was included as a predictor in the physician-discussion model. Because none of the participants who had never visited a gynaecologist reported previous physician discussion, this category was combined with visits less frequent than once every three years in the physician-discussion model to avoid complete separation and permit stable model estimation. Annual or more frequent gynaecologist visits served as the reference category. Results were expressed as adjusted odds ratios with 95% confidence intervals.

The association between age group and menstrual status was examined using a Pearson chi-square test and Cramér’s *V*. To evaluate whether menstrual status contributed information beyond the variables in the original models, three menstrual-status dummy variables were added as a second block to the linear regression and both logistic regression models, with regular menstrual cycles as the reference category. Incremental contribution was evaluated using the change in *R*^2^ and the *F*-change test for linear regression and the block likelihood-ratio chi-square test for logistic regression. One menstrual-status response contained an unclassifiable value and was treated as missing only in analyses involving menstrual status; all other analyses retained that participant’s available data.

To examine whether direct experience of menopause influenced the age-group comparisons, sensitivity analyses were conducted after excluding participants who reported menopause or menopause-inducing gynaecologic surgery. The age-group comparison of the composite awareness score was repeated using the Mann–Whitney *U* test, and the comparisons of all 10 dichotomized awareness items were repeated using Pearson chi-square tests with Cramér’s *V*. The participant with the unclassifiable menstrual-status response could not be categorized for this analysis and was also excluded.

All analyses were two-sided and exploratory or hypothesis-generating. Except for the specified Bonferroni-adjusted post hoc comparisons, no global correction for multiple testing was applied; therefore, *p*-values were interpreted as nominal, and isolated statistically significant findings were interpreted cautiously because of the risk of Type I error inflation. Nominal statistical significance was defined as *p* < 0.05, and *p*-values were reported to a maximum of three decimal places. Analyses were performed using IBM SPSS Statistics for Windows, version 31.0.1.0 (IBM Corp., Armonk, NY, USA).

### 2.5. Use of Artificial Intelligence

During the preparation of this manuscript, the authors used ChatGPT-5.4 Thinking model (OpenAI) and Paperpal for Microsoft Word, version 4.18.0, for the purposes of editorial assistance, including language refinement, text restructuring, and feedback on presentation. Access to OpenAI was paid for by the authors personally. The authors have reviewed and edited the output and take full responsibility for the content of this publication.

## 3. Results

### 3.1. Participant Characteristics

A total of 557 survey responses were received, of which 431 women aged 18–49 years were included in the analysis. Participants were divided into two age groups: 18–39 years (*n* = 163) and 40–49 years (*n* = 268), with a mean age of 39 years (SD 8). The participant characteristics are shown in Table 1. Due to study design, participant characteristics are presented descriptively and should not be interpreted as representative of all women aged 18–49 years in Latvia.

Overall, participants were highly educated, with no significant difference between groups. Compared with participants aged 18–39 years, those aged 40–49 years were more likely to be overweight or obese and reported lower physical activity levels. Menstrual status also differed significantly between the groups.

### 3.2. Item-Level Menopause Awareness

Recognition of selected menopause-related statements is shown in Table 2 and represents the primary awareness findings of the study. Overall, recognition of the term “menopause” was very high (93.7%). Participants had high recognition of hot flushes (93.0%) and of the possibility that menopause may occur before age 50 (91.6%). Recognition was moderate for menstrual cessation as a defining feature of menopause (65.0%), decline in sex hormone levels (76.6%), cessation of fertility (74.5%), and the potential for menopause to worsen health status (70.8%). In contrast, awareness that menopause may increase cardiovascular risk was limited (50.3%). Across the selected item-level statements, age-group differences were not statistically significant for nine of the ten awareness items. Evidence-aligned responses to the reverse-keyed statement that memory improvement is not associated with menopause were more frequent among participants aged 40–49 years than among those aged 18–39 years (75.7% vs. 65.6%; *p* = 0.024; Cramér’s *V* = 0.109). This nominal association was small and should be interpreted cautiously because the item-level comparisons were exploratory and were not adjusted for multiple testing.

Regarding self-perceived information about menopause-related symptoms, 32.9% of participants considered themselves informed, 55.5% partially informed, 7.2% uninformed, and 4.4% were unable to assess their level of information. The distribution did not differ between age groups (*p* = 0.522, Cramér’s *V* = 0.072).

### 3.3. Beliefs About Menopause Education and Management

Nearly three-quarters of participants (72.6%) considered it important to educate young women about the menopausal transition. Endorsement was higher among participants aged 18–39 years than among those aged 40–49 years (80.4% versus 67.9%; *p* = 0.014, Cramér’s *V* = 0.141). The association was small and should be interpreted cautiously as an exploratory, nominally significant finding.

Participants also endorsed several menopause management options as potentially helpful. Regular physical activity was endorsed as potentially helpful by 93.0% of participants, and balanced nutrition by 93.5%. Medication was endorsed by most participants (93.5%), whereas psychological support (83.8%) and social support (66.1%) were endorsed less frequently. No significant associations were observed between information sources and the helpfulness of the interventions.

### 3.4. Exploratory Composite Awareness Score

The 10-item menopause awareness score ranged from 23 to 50 (possible range 10–50), with a mean of 41.2 (SD 5.2) and a median of 41.0 (25th–75th percentiles, 38.0–45.0). Although the Shapiro–Wilk test indicated a deviation from normality (*p* < 0.001), the distribution showed only slight negative skewness (skewness = −0.378). Internal consistency was modest (Cronbach’s α = 0.688), slightly below the conventional 0.70 threshold. Sampling adequacy was acceptable (KMO = 0.704; Bartlett’s test *p* < 0.001). Exploratory factor analysis identified a dominant first factor with an eigenvalue of 2.31, explaining 23.1% of the variance. However, generally modest loadings and several low communalities indicated limited one-dimensionality.

#### 3.4.1. Factors Associated with the Composite Awareness Score

In exploratory bivariate analyses, menopause awareness scores did not differ significantly by age group, education level, or frequency of gynaecologist visits. However, participants who had previously discussed menopause with a physician had slightly higher awareness scores than those who had not (median 42.0 [38.5–45.0] vs. 41.0 [37.0–45.0]; *U* = 17,060.0, *p* = 0.041, *r* = 0.10). Participants who had previously sought information about menopause also had higher awareness scores (median 42.0 [38.0–46.0] vs. 40.0 [36.0–44.0]; *U* = 18,059.5, *p* < 0.001, *r* = 0.17). Both effect sizes were small. Awareness scores differed significantly by self-perceived level of information (Kruskal–Wallis χ^2^(2) = 34.68, *p* < 0.001, ε^2^ = 0.076). Post hoc comparisons showed that participants who considered themselves informed had higher awareness scores than both the partially informed and the uninformed, whereas the partially informed and the uninformed did not differ significantly after adjustment.

In exploratory multivariable linear regression including age group, education, prior discussion with a physician, prior information-seeking, and dummy-coded terms for BMI category, physical activity, and gynaecologist visit frequency, the overall model was statistically significant (*F* (14, 416) = 2.68, *p* < 0.001), but explained only a small proportion of variance in the composite awareness score (*R*^2^ = 0.083, adjusted *R*^2^ = 0.052). Collinearity diagnostics did not indicate problematic multicollinearity (all variance inflation factors < 1.4; all tolerance values > 0.72). Prior information-seeking was independently associated with a higher awareness score in the adjusted model (*B* = 1.85, 95% CI 0.79–2.95, *p* < 0.001). Compared with participants with normal BMI, participants in the underweight category had a lower composite-score value (*B* = −5.91, 95% CI −8.96 to −2.85, *p* < 0.001). However, this estimate should be interpreted cautiously because the underweight category contained only 12 participants and may therefore be unstable. No significant independent associations were observed for the remaining variables. Although physician discussion was associated with awareness score in the bivariate analysis, this association was not retained after adjustment. Given the study-specific nature of the composite score and the low explained variance, these regression findings should be interpreted as exploratory.

In a sensitivity model comprising 430 participants with classifiable menstrual-status data, adding menstrual status in a second block did not significantly improve the linear regression (Δ*R*^2^ = 0.009; *F*-change (3, 412) = 1.35, *p* = 0.259). The expanded model remained statistically significant (*F* (17, 412) = 2.44, *p* = 0.001; *R*^2^ = 0.091, adjusted *R*^2^ = 0.054), but none of the menstrual-status categories was independently associated with the composite awareness score. The associations with previous information-seeking (*B* = 1.84, 95% CI 0.75–2.92, *p* < 0.001) and underweight BMI (*B* = −5.80, 95% CI −8.85 to −2.74, *p* < 0.001) remained materially unchanged, and collinearity diagnostics did not indicate problematic multicollinearity.

#### 3.4.2. Sensitivity Analysis Excluding Participants with Menopausal Experience

A sensitivity analysis was conducted after excluding 33 participants who reported menopause or gynaecologic surgery resulting in menopause and one participant whose menstrual-status response could not be classified, resulting in a restricted sample of 397 participants. Composite awareness scores did not differ significantly between participants aged 18–39 years (median 41.0 [38.0–45.0]) and those aged 40–49 years (median 42.0 [38.0–45.0]; *U* = 18,509.5, *p* = 0.660, *r* = 0.020). Item-level age-group patterns also remained materially unchanged: nine of the ten comparisons were not statistically significant, and all associations were negligible or small (Cramér’s *V* = 0.006–0.119). Evidence-aligned responses to the reverse-keyed statement that memory improvement is not associated with menopause were more frequent among participants aged 40–49 years than among those aged 18–39 years (76.7% vs. 65.8%; *p* = 0.018; Cramér’s *V* = 0.119). This nominal association was small and should be interpreted cautiously because the item-level comparisons were exploratory and were not adjusted for multiple testing. Overall, excluding participants with direct menopausal experience did not materially alter the age-group comparisons. Complete item-level sensitivity results are presented in Supplementary Table S1.

### 3.5. Additional Exploratory Analyses

Composite awareness scores were higher among participants who reported obtaining menopause information from family or friends (*p* = 0.010, *r* = 0.12), the internet (*p* = 0.016, *r* = 0.12), and books or scientific literature (*p* = 0.018, *r* = 0.11). All effect sizes were small. Composite awareness scores did not differ significantly according to reported use of physician consultation, social media, television, or printed journals as information sources.

Participants aged 40–49 years had higher adjusted odds of both previous physician discussion (*aOR* = 3.23, 95% CI 1.79–5.80, *p* < 0.001) and previous menopause information-seeking (*aOR* = 3.06, 95% CI 1.89–4.94, *p* < 0.001) than those aged 18–39 years.

In separate exploratory multivariable logistic regression models, previous menopause information-seeking was independently associated with higher adjusted odds of physician discussion (*aOR* = 5.40, 95% CI 3.05–9.57, *p* < 0.001), while previous physician discussion was independently associated with higher adjusted odds of menopause information-seeking (*aOR* = 5.37, 95% CI 3.05–9.47, *p* < 0.001). Both models were adjusted for education, BMI category, physical activity level, and frequency of gynaecologist visits. In the physician-discussion model, infrequent or no gynaecologist visits (*aOR* = 0.26, 95% CI 0.10–0.67, *p* = 0.005) and approximately triennial visits (*aOR* = 0.28, 95% CI 0.15–0.55, *p* < 0.001) were associated with lower adjusted odds of physician discussion compared with annual or more frequent visits. No other modelled predictors were independently associated with either outcome.

In sensitivity analyses comprising 430 participants with classifiable menstrual-status data, adding menstrual status did not significantly improve either the information-seeking model (Δχ^2^(3) = 5.63, *p* = 0.131) or the physician-discussion model (Δχ^2^(3) = 2.06, *p* = 0.560). No menstrual-status category was independently associated with either outcome, and the principal associations remained materially unchanged.

## 4. Discussion

### 4.1. Principal Findings

In this exploratory survey of Latvian women aged 18–49 years, participants showed high recognition of commonly discussed menopausal symptoms, but substantially lower recognition of cardiovascular risk. This contrast is clinically important because the menopausal transition is increasingly recognized as a period of accelerating cardiometabolic risk and a window for early prevention [3]. Consistent with this need, 72.6% of participants considered education of younger women about the menopausal transition important.

Nine of the ten item-level comparisons showed no statistically significant age-group differences, while the item concerning whether memory improvement is associated with menopause showed one small nominal association, with a higher proportion of evidence-aligned responses among participants aged 40–49 years. This isolated finding should be interpreted cautiously because the item-level analyses were exploratory and were not globally adjusted for multiple testing. The composite awareness score also did not differ between age groups, and exclusion of participants who reported menopause or menopause-inducing gynaecologic surgery did not materially alter either the item-level pattern or the composite-score comparison. Thus, although participants aged 40–49 years were more likely to report previous information-seeking and physician discussion, this greater exposure was not accompanied by higher overall measured awareness. Likewise, the absence of associations with education level and gynaecologist visit frequency should not be interpreted as evidence that these factors are irrelevant to menopause literacy. General educational attainment does not necessarily capture menopause-specific health knowledge, and gynaecologist-visit frequency before midlife does not indicate whether menopause was discussed or capture the content or quality of any counselling provided [1,16].

Exploratory analyses showed that prior menopause information-seeking was independently associated with higher awareness scores, whereas age group and prior physician discussion were not. Underweight BMI was also associated with a lower awareness score, but this estimate was based on only 12 participants and may be unstable and should be regarded as hypothesis-generating.

### 4.2. Comparison with Previous Evidence

The high recognition of hot flushes in the present sample (93.0%) closely resembled the 92.2% reported in a nationally stratified population survey in Wales [5]. Nevertheless, 31.1% of participants in the Welsh survey reported low menopause knowledge, with lower knowledge particularly evident in younger age groups [5]. Among UK women younger than 40 years, more than 80% reported no or only limited menopause knowledge; women older than 30 years were more likely to use official websites and scientific literature, whereas younger women relied more frequently on family members [13]. Similarly, a UK survey of women older than 40 years found that most had been uninformed or only partly informed about menopause before age 40, with independent websites and friends among the most common information sources [4]. Surveys of perimenopausal and postmenopausal women also found that formal menopause education was uncommon and that information-seeking frequently began only after symptoms developed [12,15]. Collectively, these findings indicate that recognition of well-known symptoms can coexist with limited broader preparation for menopause. Direct numerical comparisons should remain cautious because the studies differed in their populations, age ranges, sampling methods, questionnaires, and definitions of menopause knowledge or awareness. Moreover, these surveys did not consistently assess awareness of long-term cardiovascular risk using directly comparable items. The finding that only 50.3% of participants in the present study recognized the association between menopause and cardiovascular risk therefore provides complementary evidence of a specific awareness gap. Menopause education should begin before the menopausal transition and extend beyond symptom recognition to include cardiometabolic health and opportunities for early prevention [1,3].

The clinical relevance of this cardiovascular-awareness gap is reinforced by life-course evidence linking reproductive history and reproductive ageing with cardiometabolic outcomes. An umbrella review of 13 reviews encompassing 283 original studies and more than 6.8 million participants found that early menarche was associated with increased risks of obesity, hypertension, metabolic syndrome, type 2 diabetes or impaired glucose tolerance, and all-cause mortality [17]. In the Million Women Study of 740,628 postmenopausal women, mean BMI among parous participants was approximately 1% lower for each six months of lifetime breastfeeding [18]. A cross-sectional analysis of 413,802 women from 15 countries found that later age at first birth was associated with lower BMI and systolic blood pressure in both premenopausal and postmenopausal women, whereas associations involving parity and fasting glucose varied by outcome and menopausal status [19].

Comparable associations have been reported in Central and Eastern European populations. Among 503 Slovak women aged 39–65 years, menarche at age 11 years or younger was associated with higher odds of midlife obesity (*OR* = 2.27, 95% CI 1.35–3.81, *p* = 0.002), while age at menarche was separately associated with obesity-associated hypertension. Among parous women, breastfeeding was associated with lower odds of obesity than never breastfeeding [20]. Among 2419 young adult Polish women aged 19–24 years, earlier menarche was likewise associated with higher BMI, waist circumference, and waist-to-height ratio, with the strength of these associations differing between birth cohorts [21]. Longitudinal SWAN data further showed that the rate of fat gain nearly doubled at the onset of the menopausal transition, while lean mass began to decline; these changes continued until approximately two years after the final menstrual period and occurred independently of chronological ageing [22].

The relevance of reproductive ageing may also extend beyond cardiometabolic outcomes. In another cross-sectional study of 503 Slovak women, miscarriage history was associated with early waking followed by poor sleep, the number of children was associated with mood-related symptoms, and postmenopausal women were more likely to report severe vaginal dryness than premenopausal and perimenopausal women [23]. Although these observational findings do not establish causality, they support a life-course perspective connecting reproductive history and menopausal status with cardiometabolic, psychological, sleep-related, and urogenital health. They therefore provide a rationale for menopause education and preventive counselling that address modifiable cardiovascular risk factors and broader midlife health, rather than immediate symptoms alone.

The association between previous information-seeking and higher awareness is consistent with evidence that women frequently obtain menopause information through self-directed and informal sources. In a UK survey of women younger than 40 years, women older than 30 reported more proactive information-seeking and greater use of official websites, social media, and the scientific literature than women younger than 20, while family and friends were common information sources across age groups [13]. Nevertheless, the absence of an independent association with physician discussion should not be interpreted as evidence that clinical counselling is ineffective. In a Norwegian general-practice survey of 625 women, 59% wanted information about menopause; among those who wanted information, but whose general practitioner had not addressed the subject, 81% wanted the practitioner to do so, whereas only 10% reported that their practitioner had initiated such a discussion [24]. Our binary measure recorded only whether a previous physician discussion had occurred and did not capture its timing, content, duration, or quality. Moreover, because the study was cross-sectional, we cannot determine whether information-seeking improved awareness, whether more aware participants were more likely to seek information, or whether both reflected a broader tendency toward health engagement. Similarly, the loss of statistical significance for physician discussion after adjustment may reflect overlapping help-seeking behaviours rather than the irrelevance of clinical counselling.

Directly comparable published evidence on menopause awareness in Baltic populations remains limited, precluding robust comparison of the present findings with Estonia, Lithuania, or other neighbouring countries. Broader European research suggests that menopause-related awareness and information-seeking may be influenced by previous education, general health literacy, access to understandable language-specific information, communication with healthcare professionals, and sociocultural attitudes toward menopause [1,5,13,24]. These factors may also be relevant in Latvia, but they were not measured in the present study and therefore remain hypotheses rather than explanations for the observed findings. Accordingly, the results describe this Latvian sample and should not be interpreted as evidence of a relative national awareness deficit or shortcomings in the Latvian healthcare system.

### 4.3. Clinical Implications

These exploratory findings identify potential priorities for clinical practice and public health education rather than demonstrating the effectiveness of any particular intervention. Menopause education could begin before women reach the menopausal transition, consistent with the finding that 72.6% of participants considered education of younger women important and with previous studies documenting limited education before midlife [4,13,14]. Educational content should retain information about recognizable symptoms while expanding coverage of cardiovascular and metabolic health, including blood-pressure control, smoking cessation, healthy body composition, and physical activity [3]. Potential delivery channels include age-appropriate education in schools and universities, primary care, gynaecology, public-health communication, and reliable online resources, reflecting the different information preferences reported across previous surveys [13,14,24,25]. The absence of an independent association between previous physician discussion and awareness does not establish that clinical counselling is ineffective because the survey did not assess the timing, content, or quality of those discussions. Public-facing materials should also distinguish interventions supported by evidence from approaches with uncertain efficacy [1,26]. Given the methodological limitations identified in the present study, future Latvian-language educational materials and awareness instruments should undergo structured cultural adaptation, cognitive pretesting, and psychometric evaluation. Their effects on awareness or knowledge, risk perception, care-seeking, and health behaviour should then be assessed prospectively in more representative samples.

### 4.4. Limitations

Several limitations constrain generalizability and causal interpretation. The cross-sectional design precludes conclusions about temporality or causality, including whether information-seeking increased awareness or whether participants with greater awareness were more likely to seek information. Recruitment through paid Facebook posts constituted non-probability convenience sampling and introduced potential selection bias related to platform use, voluntary participation, and interest in menopause. The relatively highly educated sample may therefore not represent all women aged 18–49 years in Latvia. Unique click-through and form-access data were unavailable, preventing calculation of a conventional response rate. Because IP tracking, login restrictions, or comparable safeguards were not used, duplicate submissions could not be completely excluded.

Eligibility and all participant characteristics were self-reported and were not independently verified. All survey questions were mandatory; therefore, no partially completed questionnaires were recorded, but mandatory responses may also reduce nuance in participant answers.

The investigator-designed questionnaire was informed by English-language questionnaires and adapted into Latvian by native Latvian-speaking authors, with subsequent review by native Latvian-speaking gynaecologists. However, no formal content-validity assessment, forward–back translation, structured cross-cultural adaptation, cognitive pretesting, pilot testing, or pre-administration psychometric validation was performed. These omissions may limit content validity and cross-cultural comparability, and questionnaire-derived findings should therefore be regarded as exploratory.

Dichotomization of Likert responses also combined neutral and evidence-discordant responses and removed distinctions in response intensity. This information loss may have reduced the sensitivity of item-level comparisons and obscured subtler differences across the original ordinal categories. Accordingly, non-evidence-aligned responses should not be interpreted uniformly as incorrect, and nonsignificant comparisons do not exclude smaller differences in response distributions. These item-level findings should therefore be interpreted as descriptive and hypothesis-generating.

The composite awareness score was constructed post hoc, and the same sample was used for item selection and subsequent assessment of internal consistency and dimensional structure. Because the final item set was selected partly to improve Cronbach’s α, this process introduced selection-related bias and may have produced optimistic or sample-specific estimates of internal consistency and factor structure. The modest α and limited one-dimensionality further indicate that the score should not be regarded as a validated psychometric measure. In addition, the reverse-keyed items concerning memory improvement and improved libido may have been more difficult to interpret without cognitive pretesting or pilot testing. This may have contributed to their lower evidence-aligned response rates and to the modest internal consistency of the score; responses to these items should therefore not be interpreted as reflecting lack of awareness alone. Associations involving the composite score should be considered hypothesis-generating and require independent evaluation using a prespecified item set.

Except for Bonferroni-adjusted post hoc comparisons following significant Kruskal–Wallis tests, no global correction for multiple testing was applied. The numerous item-level and exploratory analyses therefore increased the risk of Type I error, and isolated nominally significant findings should be interpreted cautiously. Some predictor categories were also sparsely represented, particularly underweight BMI (*n* = 12) and daily physical activity (*n* = 10), potentially reducing the precision and stability of their regression estimates. The association between underweight BMI and a lower composite awareness score may consequently be sample-specific and requires confirmation. Although participants with direct menopausal experience may have possessed experiential awareness, excluding these participants did not materially alter the age-group comparisons. Finally, the low explained variance of the regression model indicates that relevant determinants—including previous menopause education, family experience, broader health literacy, and cultural attitudes—were not fully captured. Future studies should use more representative recruitment, cognitively pretested and culturally validated Latvian instruments, and prespecified composite measures evaluated in independent samples.

## 5. Conclusions

These exploratory findings support the development and evaluation of evidence-based menopause education in Latvia that begins before the menopausal transition and integrates symptom recognition with cardiometabolic risk awareness.

Given the non-probability sample and study-specific, non-validated measures, future studies should use representative sampling, validate culturally adapted Latvian instruments, and prospectively evaluate educational interventions.

## Figures and Tables

**Table 1:** Participant characteristics by age group.

Variable	Total (*n* = 431)	Age Group 18–39 Years (*n* = 163)	Age Group 40–49 Years (*n* = 268)	* *p*-Value	Cramér’s *V*
Higher education	313 (72.6%)	118 (72.4%)	195 (72.8%)	0.934	0.004
BMI category				<0.001	0.292
Underweight	12 (2.8%)	8 (4.9%)	4 (1.5%)		
Normal weight	197 (45.7%)	101 (62.0%)	96 (35.8%)		
Overweight	137 (31.8%)	35 (21.5%)	102 (38.1%)		
Obesity	85 (19.7%)	19 (11.7%)	66 (24.6%)		
Physical activity level				0.014	0.170
No	149 (34.6%)	41 (25.2%)	108 (40.3%)		
Irregularly, rarely	123 (28.5%)	49 (30.1%)	74 (27.6%)		
1–2 times per week	96 (22.3%)	41 (25.2%)	55 (20.5%)		
3–4 times per week	53 (12.3%)	27 (16.6%)	26 (9.7%)		
Daily	10 (2.3%)	5 (3.1%)	5 (1.9%)		
† Menstrual status				0.001	0.190
Regular menstrual cycle	284 (66.0%)	116 (71.2%)	168 (62.9%)		
Irregular menstrual cycle	82 (19.1%)	32 (19.6%)	50 (18.7%)		
Menopause or menopause due to gynaecologic surgery	33 (7.7%)	2 (1.2%)	31 (11.6%)		
‡ Hormonal/reproductive condition	31 (7.2%)	13 (8.0%)	18 (6.7%)		

* *p*-values represent comparisons between age groups for each overall characteristic. Pearson’s chi-square tests were used for higher education, BMI category, physical activity level, and menstrual/menopausal status. Effect sizes are reported as Cramér’s *V*. † Menstrual/menopausal status was available for 430 participants; one submitted response could not be classified into the prespecified categories. ‡ Hormonal/reproductive condition included hormone-related or reproductive states affecting menstrual status.

**Table 2:** Item-level menopause awareness and recognition of selected menopause-related statements.

Statement	Median (Q1–Q3)	Total (*n* = 431)	Age Group 18–39 Years (*n* = 163)	Age Group 40–49 Years (*n* = 268)	* *p*-Value	Cramér’s *V*
Menstrual cessation corresponds to menopause	4 (3–5)	280 (65.0%)	99 (60.7%)	181 (67.5%)	0.151	0.069
A decline in sex hormone levels corresponds to menopause	4 (3–5)	330 (76.6%)	118 (72.4%)	212 (79.1%)	0.111	0.077
Fertility cessation corresponds to menopause	4 (3–5)	321 (74.5%)	119 (73.0%)	202 (75.4%)	0.585	0.026
Menopausal symptoms may appear before age 50	5 (5–5)	395 (91.6%)	150 (92.0%)	245 (91.4%)	0.825	0.011
Hot flushes are associated with menopause	5 (4–5)	401 (93.0%)	147 (90.2%)	254 (94.8%)	0.069	0.088
Vaginal dryness is associated with menopause	5 (4–5)	344 (79.8%)	131 (80.4%)	213 (79.5%)	0.823	0.011
Menopause can worsen health status	4 (3–5)	305 (70.8%)	115 (70.6%)	190 (70.9%)	0.939	0.004
Menopause can increase cardiovascular risk	4 (3–5)	217 (50.3%)	90 (55.2%)	127 (47.4%)	0.115	0.076
† Memory improvement is not associated with menopause	5 (3–5)	310 (71.9%)	107 (65.6%)	203 (75.7%)	0.024	0.109
† Improved libido is not associated with menopause	4 (3–5)	258 (59.9%)	106 (65.0%)	152 (56.7%)	0.088	0.082

* Values are shown as *n* (%) of participants providing evidence-aligned responses unless otherwise indicated. Median values are based on the original 5-point responses for positively keyed items. For the two reverse-keyed items, values were reverse-coded. Higher displayed values therefore consistently indicate greater awareness. *p*-values were calculated using the χ^2^ test for comparison between age groups. † Reverse-keyed item. Original responses of 1–2 were considered evidence-aligned and were reverse-coded before calculating the composite awareness score. Statement labels are presented in the corresponding evidence-aligned direction and do not reproduce the original questionnaire wording.

## Data Availability

The data supporting the conclusions of this article can be made available by the authors upon request.

## Supplementary Materials

The following supporting information can be downloaded at https://www.mdpi.com/article/10.3390/jcm15166295/s1. Supplementary Table S1: Sensitivity analysis of item-level menopause awareness by age group after excluding 33 participants who reported menopause or gynaecologic surgery resulting in menopause and one participant with an unclassifiable menstrual-status response (*n* = 397).

## Abbreviations

The following abbreviations are used in this manuscript:

BMI	Body Mass Index
CROSS	Checklist for Reporting of Survey Studies
CHERRIES	Checklist for Reporting Results of Internet E-Surveys
KMO	Kaiser-Meyer-Olkin
