# Supplementary material for: Awareness of Menopause and Health Risks Among Reproductive-Age Women in Latvia
Source: J Clin Med. 2026 Aug 14;15(16):6295. doi: 10.3390/jcm15166295 (PMC13513038; doi:10.3390/jcm15166295)
Supplement: Supplementary file 1 [file jcm-15-06295-s001.zip › jcm-4419327-supplementary.pdf]

Article

Awareness of Menopause and Health Risks Among Reproductive-Age Women in Latvia

Jānis Jurkāns *, Jeļizaveta Kolosovska and Elizabete Ārgale

Department of Obstetrics and Gynaecology, Riga Stradins University, LV-1007 Riga, Latvia
* Correspondence: janis.jurkans.med@gmail.com

Supplementary Table S1.

Sensitivity analysis of item-level menopause awareness by age group after excluding 33 participants who reported menopause or gynaecologic surgery resulting in menopause and one participant with an unclassifiable menstrual-status response (n = 397).

Statement	Median (Q1-Q3)	Total (n = 397)	Age group 18–39 years (n = 161)	Age group 40–49 years (n = 236)	* p–value	Cramér's V
Menstrual cessation corresponds to menopause	4 (3–5)	257 (64.7%)	97 (60.2%)	160 (67.8%)	0.122	0.078
A decline in sex hormone levels corresponds to menopause	4 (4–5)	301 (75.8%)	116 (72.0%)	185 (78.4%)	0.147	0.073
Fertility cessation corresponds to menopause	4 (3–5)	295 (74.3%)	117 (72.7%)	178 (75.4%)	0.538	0.031
Menopausal symptoms may appear before age 50	5 (5–5)	363 (91.4%)	148 (91.9%)	215 (91.1%)	0.773	0.014
Hot flushes are associated with menopause	5 (4–5)	368 (92.7%)	145 (90.1%)	223 (94.5%)	0.096	0.084
Vaginal dryness is associated with menopause	5 (4–5)	316 (79.6%)	129 (80.1%)	187 (79.2%)	0.830	0.011
Menopause can worsen health status	4 (3–5)	280 (70.5%)	113 (70.2%)	167 (70.8%)	0.902	0.006
Menopause can increase cardiovascular risk	3 (3–5)	195 (49.1%)	88 (54.7%)	107 (45.3%)	0.068	0.092
† Memory improvement is not associated with menopause	5 (3–5)	287 (72.3%)	106 (65.8%)	181 (76.7%)	0.018	0.119
† Improved libido is not associated with menopause	4 (3–5)	239 (60.2%)	105 (65.2%)	134 (56.8%)	0.092	0.085

* Values are shown as n (%) of participants providing evidence-aligned responses unless otherwise indicated. Medians and Q1–Q3 represent the median and first and third quartiles of the five-point Likert scores. For positively keyed items, the original scores were retained. For the two reverse-keyed items, scores were reverse-coded so that higher displayed values consistently indicated greater awareness. Age-group comparisons of evidence-aligned responses were performed using Pearson's chi-square tests, with Cramér's V reported as the corresponding effect-size measure. All p-values are nominal; no global correction for multiple testing was applied. † Reverse-keyed item. Original responses of 1–2 were classified as evidence-aligned and were reverse-coded before calculation of the composite

awareness score. Statement labels are presented in the corresponding evidence-aligned direction and do not reproduce the original questionnaire wording.
